# Voices from the minority: Understanding the acculturative experiences of British Shia Muslims

**DOI:** 10.1111/papt.70058

**Published:** 2026-03-27

**Authors:** Mahdiyah Datoo, Sanaa Kadir

**Affiliations:** ^1^ University of Leicester Leicester UK

**Keywords:** acculturation, identity, intersectionality, mental health, Muslims

## Abstract

**Objectives:**

The aim of this research was to understand the acculturative experiences of British Shia Muslims, with hopes for practitioners to better understand how to support this population.

**Design:**

Qualitative methodology was used, utilising semi‐structured interviews. Braun and Clarke's (*Thematic analysis: A practical guide*, Sage Publications Limited, 2022) reflexive thematic analysis was chosen to analyse the data and identify themes within 15 interview transcripts.

**Methods:**

Recruitment took place through mosques, with inclusion criteria involving British Shia Muslim participants over 18 years, who had lived in England for a minimum of 5 years. First‐, second‐ and third‐generation participants were recruited.

**Results:**

Five themes were identified, sitting under the main overarching theme, ‘navigating through multiple worlds’, which depicted acculturation as a fluid, complex journey, consistent with Schwartz et al.'s (*American Psychologist*, 2010, 65, 37) multidimensionality model of acculturation. These themes included: do I belong here, with subthemes of conflicting cultural values and who am I, the emotional toll of acculturation, with subthemes of negotiating authenticity and Muslim therapeutic support, negotiating faith in a secular society, living with layered discrimination, with a subtheme of Shia‐specific discrimination and, finally, holding each other up: sources of strength.

**Conclusions:**

This research demonstrates the multidimensional and complex journey of acculturation for British Shia Muslims. Through this, there is an emphasis on understanding more about the experiences of minoritised communities to improve their mental health support. The clinical implications to support this are diversifying the workforce, enhancing cultural competence and humility, adopting an intersectional approach and addressing discrimination.

## INTRODUCTION

Within clinical psychology, holding an intersectional lens is essential to understanding an individual's unique experiences and their multiple, intersecting identities (Tang et al., 2020). Kelly et al. (2021) explain that intersectionality understands that individual social categorisations such as race and religion overlap and intersect, reflecting forms of oppression and privilege. Intersectionality can be used as an analytic tool to challenge such social inequalities within these systems of oppression and privilege (Grzanka et al., 2020). Anwar (2022) further explains that intersectional theory accounts for individuals facing multiple sources of oppression and being aware of this when working psychologically is crucial in supporting someone's mental wellbeing. Rosenthal (2016) suggests that one way to address intersectionality within psychology is through collaboration with all communities to work together and build partnerships. When considering intersectionality within immigrant and minoritised communities, acculturative experiences and their impact on mental health can be investigated.

### Acculturative experiences

Acculturation refers to the changes that occur when individuals interact with people, groups or social influences that are dissimilar to their own (Gibson, 2001). Acculturation is known to encompass the changes in all groups following intercultural contact (Kunst, 2025). It is important to recognise its impact within multicultural societies, as acculturative experiences involve a reciprocal exchange of different cultural groups (Schwartz et al., 2010). Exploring acculturation can involve learning about the values and beliefs of immigrant families, including behaviours that affect health (Unger et al., 2000). Berry (2003) has stated that acculturation is one of the most researched concepts in immigration literature, recognised as one of the strongest contributors to understanding psychological states. This may be due to acculturative stress, a term introduced by Berry (2007) as an alternative to ‘culture shock’. Sirin et al. (2012) conducted a longitudinal study involving 337 immigrant adolescents from America and found that acculturative stress was a significant risk factor for mental health difficulties. This stress increased vulnerability to psychological challenges, highlighting the need for appropriate psychological support (Sirin et al., 2012). Furthermore, Cavdar et al. (2021) conducted acculturative research in a sample of 220 Turkish adolescents in England and found that those with stronger levels of ethnic identity were less likely to assimilate to the British society, leading to better reported levels of mental health. Cavdar et al.'s (2021) findings demonstrate the links between acculturative assimilation, ethnic identity and mental health.

**FIGURE 1:**
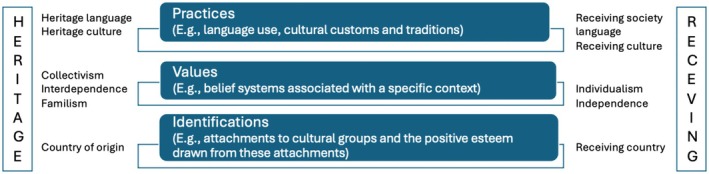
Multidimensionality of acculturation (Schwartz et al., 2010). This figure is an adapted version of Schwartz et al.'s (2010) model.

When thinking about theoretical models of acculturation, we can look to Schwartz et al.'s (2010) Multidimensionality of Acculturation (Figure 1). This model understands acculturation as a complex phenomenon, which involves a dynamic process where individuals must negotiate their practices, values and identifications between their origin culture and the culture of the country they are residing in. They explain that these three domains can shift independently and at different times, providing a fluid understanding of acculturative experiences. The multidimensional lens within this model distinguishes that acculturative experiences can be shaped by wider sociocultural factors, such as community, discrimination and intergenerational differences. Due to this, Schwartz et al.'s (2010) model will be used to understand acculturation within this research.

Another acculturation framework that dominates the literature in this area comes from Berry (1997), who identified four acculturation categories. The first is integration, which refers to the participation of an immigrant's host culture as well as retainment of their own. The second is assimilation, describing someone participating within the host culture and rejecting their own. Third is separation, where someone retains their cultural norms and rejects the host culture. Fourth is marginalisation, which involves the rejection of both the host culture as well as their own (Berry, 1997). Schwartz et al. (2010) argue that Berry's (1997) framework is reductionist, as it does not account for the complexity of acculturation that involves concepts such as ethnicity, cultural similarity and discrimination.

### British Muslim mental health

Youngman (2024) has argued that the absence of religious and cultural awareness in practitioners significantly impacts the support that British Muslims receive, further stating that the National Health Service (NHS) is failing Muslims due to the lack of consideration of culture and faith. Hekmoun and Abrar's (2024) report, ‘Faith in Mental Health’, which considered mental health in Muslim communities, noticed significant discrepancies between Muslim mental health outcomes compared to those of other faiths. Between 2021 and 2022, 46,000 Muslims were referred to NHS Talking Therapies, but only 2.65% completed treatment, compared with 40% who stated no religious faith (NHS, 2022). Overall, the report noted that Muslims felt their faith was repeatedly ignored by professionals and was often viewed negatively. The above statistics demonstrate a clear need for improvement of mental health services for this demographic as there is an evident mismatch in the services currently provided. The report urged for practitioners to better understand the mental health of Muslim communities, and this project intends to be a starting point for this.

The above information can be understood further through the lens of acculturation. Ahmed and Reddy (2007) explained that the challenges Muslims face when acculturating, such as intense discrimination and economic changes, can lead to high levels of psychological distress, including low self‐esteem, feelings of hopelessness and depression. Through this research, we can understand that acculturation for Muslims may be different to the experiences of others. The lack of awareness of Muslim acculturative hardships from services could explain this population's resistance to help‐seeking, which has been noted in the literature (McLaughlin et al., 2022; Pilkington et al., 2011; Tanhan & Young, 2021). Tanhan and Young's (2021) literature review found that institutional barriers were a significant factor within this, as Muslims did not feel there were appropriate services they could engage with. It was stressed by the authors of the literature reviewed that spiritually and culturally appropriate services must be developed for the Muslim population (Tanhan & Young, 2021).

This research shows the important link between understanding acculturation and its impact on mental health. For example, Tineo et al. (2021) examined the mental health impacts of discrimination among 2015 American Muslim college students. Their findings showed statistically significant indirect effects of perceived discrimination on depression and anxiety, mediated by acculturative stress. This research allows us to contextualise the importance of looking at acculturation and how it may affect mental health. By practitioners understanding more on Muslim acculturative experiences, cultural understandings could be understood further, the unique stressors can be identified, thus increasing the effectiveness of mental health support.

### The Shia Muslim community

This research project will specifically focus on the experiences of Shia Muslims. Shi'ism is a sect of Islam, understood to be the minority within the UK population, with 3.8 million Muslims in England and 5% of these identifying as Shia (BBC Teach, 2025). As identifying as a British Shia Muslim myself, I personally reflect on the challenges that came with growing up in a Muslim household within a western world. Similar reports have been found within literature, demonstrating that Muslims have competing demands across their families, friends, the Muslim community, the wider society and religion (Stuart & Ward, 2011). Additionally, global research tells us that the Shia Muslim population face high levels of adversity. Musa and Tan's (2017) research reported that Shia Muslims in Malaysia experience ill‐treatment and hostility, which was supported by the state. Furthermore, Watch and Wilcke (2009) shared that Shia‐specific discrimination in Saudi Arabia is on the rise, stating that this tended to be government led. Syed and Ali (2020) also identified a ‘pyramid of hate’ directed at Shia Muslims in Pakistan, which begun at a societal level and infiltrated into their professional lives, often leading to harassment, violence and broader societal exclusion. There was no identified research on Shia populations in England, further stressing the importance of this research.

There are various cultural differences that set Shia Muslims apart from other Muslim sects. The most common difference found within the literature mentions the importance of the historical event of ‘Karbala’, which is commemorated on a yearly basis with the 10 days of ‘Ashura’ (Jan, 2023). This is a time of mourning for Shia's through remembrance of a battle that took place in 680 CE where Imam Husayn, the grandson of the Holy Prophet Muhammad, a prominent Islamic figure, was martyred. During the 10 days of Ashura, there are regular gatherings where Shia Muslims will hear the stories that took place during the battle of Karbala, recognising the sacrifices and struggles of Imam Husayn and his family. The ‘Ahlul Bayt’ (family of the Prophet) also hold a lot of importance within the Shia sect, especially Imam Ali and Bibi Fatemah, as they are looked to as divinely guided exemplars (Momen, 1985). Imam Ali is known to be the successor following the Holy Prophet, which is debated within other Islamic sects.

Scharbrodt (2019) has stated that Shia Muslims are ‘a minority within a minority’, signifying the double marginalisation of this group. Therefore, the complexities of acculturation may be enhanced for Shia Muslims. They may need to manage the ideologies of the host societies' western world, alongside their identity as Shia Muslims within the Islamic world. Furthermore, Scharbrodt (2020) highlighted the anti‐Shia hostility that arose following the introduction of the Islamic State of Iraq and Syria (ISIS) in 2014, thus emphasising the concept of ‘the other within the other’ (Takim, 2009).

Moreover, as recognised, there is a dearth of research generally on the British Shia Muslim population. Though not based on acculturation, there is some literature discussing Shia Muslims' attitudes towards mental health in America. Hussain's (2022) cross‐sectional quantitative research identified treatment barriers across 256 Shia Muslims, with participants reporting a mistrust of the mental health care systems, stating worries of being part of an ‘agenda’ that could stray them away from their values. Another barrier recognised was the lack of cultural competence within services, with participants feeling that professionals would not appreciate their difficulties due to a lack of understanding of their faith. By increasing cultural understanding through learning acculturative experiences, this could be improved. Cheon et al. (2020) has explained that America and England are both considered Western, Educated, Industrialised, Rich and Democratic (WEIRD) countries. As there is not enough research on British populations, we may be able to assume that similar findings could be applied to a sample of British Shia Muslims. However, this requires exploration as there are differences in various aspects between American and England. This is what this research aims to do.

### Rationale and aims for project

The aim of this research was to understand the acculturative experiences of British Shia Muslims, to further apprehend its role in impacting mental health. There is a dearth of literature within this field, resulting in minimal knowledge and recognition on how acculturation may impact the mental health of British Shia Muslims and the support they receive. It is hoped that this exploratory research will support mental health services and practitioners to increase their cross‐cultural knowledge, widen their intersectional lens and better understand how to support this population.

## METHOD

### Study design

A qualitative design using semi‐structured interviews was utilised, in line with the research aims. As the aims were to investigate the experiences of acculturation for individuals, a qualitative design supports this by collecting rich, in‐depth data to understand meanings (Willig, 2008). Reflexive thematic analysis (RTA, Braun & Clarke, 2022) was the chosen analytic process to identify themes within the interview transcripts. Patient and Public Involvement and Engagement was considered through a Service User Reference Group (SURG). A summary of the project was sent to SURG in which they provided feedback on whether the project held relevance and clarity.

### Participants

Fifteen participants were recruited for this project. Individual participant demographics can be found in Table 1, demonstrating the range of ages, genders and generational statuses. The inclusion criteria for recruitment involved adult participants (over 18 years of age) who had resided in England for a minimum of 5 years. The 5‐year limit was based on research explaining that those who acquire settlement do so within the first 5 years of receiving their initial visa to live in England (Walsh & Jorgensen, 2024). Additionally, 5 years gives people the opportunity to adjust and increase their experience with acculturation. No language restrictions were applied, as this may have led to the exclusion of people who have different, unique acculturative experiences. However, all participants spoke English, with 93% referring to English as their first language. Participants had to identify as part of the Shia sect of Islam, and recruitment of first, second and third generations was attempted. However, no third‐generation participants came forward. In accordance with Lessard‐Phillips and Li (2017), first‐generation immigrants refer to those who were born elsewhere and have migrated to England, second‐generation immigrants include those who have a parent born elsewhere and third‐generation immigrants are those with both parents born in England and a grandparent born elsewhere. The variety of generations was to allow for a range of experiences, offering insight from various perspectives. Van Der Zee and Van Oudenhoven (2022) have also explained that acculturative experiences are not specific to immigrants, as it is a process of cultural change, justifying inclusion for second‐ and third‐generation participants. Exclusion criteria involved those under the age of 18 who did not identify as Shia and who had resided in England for less than 5 years. Participants who had a relationship with the researcher were also excluded from this research.

**TABLE 1:** Individual participant demographics.

Pseudonym^a^	Gender	Age	Generational status	Years living in England	Country of origin	Parents country of origin
Adam	Male	45	Second	45	England	Uganda
Ahmed	Male	37	Second	37	England	Tanzania and Kenya
Alaina	Female	40	First	14	Tanzania	Uganda and Tanzania
Ali	Male	49	First	27	Tanzania	Tanzania and Somalia
Ameera	Female	22	First	5	Kenya	Kenya
Hawa	Female	23	First	6	Tanzania	Tanzania
Inayah	Female	20	First	16	Pakistan	Pakistan
Iqra	Female	40	Second	40	England	Tanzania
Jawad	Male	19	Second	19	England	India
Mariah	Female	22	First	15	Pakistan	Pakistan
Moe	Male	24	First	17	Kenya	Kenya
Nadia	Female	24	Second	24	England	Tanzania
Sarah	Female	48	First	24	Somalia	Somalia
Zain	Male	51	First	40	Tanzania	Tanzania
Zara	Female	46	Second	46	England	Tanzania

^a^ Pseudonyms were used to protect participant anonymity.

### Interview questions

A semi‐structured interview was created in accordance with the research aims. This approach was deemed the most appropriate as it provided a guide for interviewees but offered opportunities for participants to expand on areas important to them. The interview schedule asked demographic questions, such as participants' ethnicity, age, number of years they have lived in England and generation status. Questions inquired about participant experiences of living in England, religious contribution, mental health experiences and discrimination.

### Procedure

Participants were recruited through Shia mosques within the East and West Midlands. A recruitment poster was created, distributed and shared via email chains and social media apps (WhatsApp, Instagram, Facebook). Following contact from participants, inclusion criteria were checked, and the Consent Form, Participant Information Sheet and Privacy Notice were sent. Following receipt of the signed consent form, interviews were arranged via Microsoft Teams, with video or audio call options.

At each interview, research aims and ethical issues were discussed, and verbal consent was gained. Interviews lasted for approximately 60 min and followed the semi‐structured interview schedule. The recordings were transcribed by the researcher in accordance with RTA (Braun & Clarke, 2022), within 28 days of the interview. Through this process, data were pseudonymised and became unidentifiable. Participants were able to withdraw up until the point of transcription. Following the end of the interview, a debrief sheet was emailed to each participant, along with a £15 voucher as compensation for their time.

Braun and Clarke's (2022) RTA was the chosen analysis method. Before going forth with each phase of their process, chapters from their practical guide (Braun & Clarke, 2022) were read and adhered to. Their process entailed familiarisation of the data, doing coding, generating initial themes, reviewing themes, defining and reviewing themes and, finally, producing the report. A theoretical, inductive approach was taken as the analysis was driven by the research question. Previous themes from research around acculturation were considered but did not lead the analytic process. Analysis began at a semantic level but progressed to a latent level. As this project was a relatively new area of research, RTA was favoured to draw out themes that reoccurred across the dataset, in an attempt to understand the group of participants as a whole. This was not an attempt to generalise the data, as the importance of individual experience was considered; however, it allowed meanings and experiences to be appreciated.

### Position of the researcher

An interpretivist position was taken throughout this project, which acknowledges that the interpretation of experiences constructs knowledge (Hiller, 2016). Hudson and Ozanne (1988) have stated that an interpretivist researcher enters the project with some insight of the research context but is open to new knowledge that develops throughout the study. This evidences the vital role of an active researcher, as themes identified are influenced by the researcher's experiences.

### Ethical issues

University ethical approval was obtained for this research. Participants were sent the consent form and participant information sheet prior to arranging the interview. At the start of interviews, ethical issues were explicitly discussed, and participants were asked whether they had any questions. Conversations included: confidentiality and its limits, the process of storing the recording of the interview, the anonymity process through transcription and information around withdrawing from the study. Verbal consent was gained following this discussion. Email addresses were used for communication and setting up the Microsoft Teams call. Participants who opted in for a lay summary of the results provided consent for their email addresses to be stored securely and separately to the interview transcripts.

As this research project took place within my own community, attention had to be paid to the appropriateness of participants. Participants who I had a relationship with were excluded to ensure formalities were adhered to and to prevent demand characteristics or social desirability bias from emerging. Furthermore, an awareness was developed on how my intersectionality may have impacted the interpretation of data. To manage this, a university research supervisor coded a subset of transcriptions independently to ensure the codes and themes were driven by the data.

## RESULTS

**FIGURE 2:**
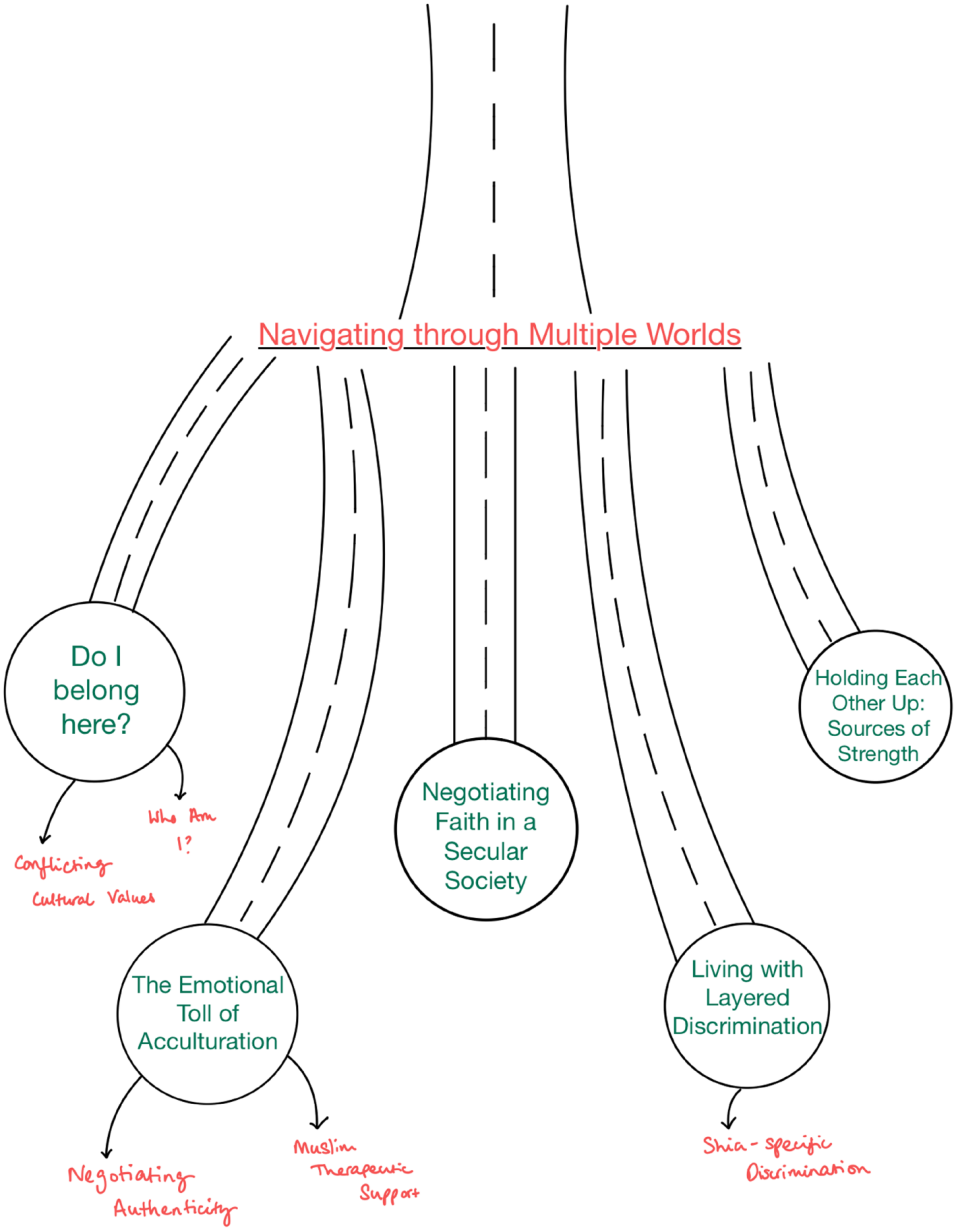
Thematic map of themes and subthemes found through reflexive thematic analysis.

Through the interviews and analysis process, it was understood that participants were on a journey, which led to the overarching theme being ‘navigating through multiple worlds’. The specific themes and subthemes encompassed within this can be found in Figure 2. The overarching theme represented that participants' interaction with themes was fluid, and they each travelled to different themes at different times, at their own pace. Furthermore, it demonstrated that acculturative processes are not singular, but participants endure multiple twists and turn on their journey through acculturation. There are several roads that they venture down and may revisit throughout their acculturative process. The themes and subthemes encompassed within this can be found below.

### Do I belong here?

Across this theme, participants shared a lack of belonging to England, feeling as though they did not fit in anywhere; ‘I remember being so left out…feeling like…I don't belong here…’ [Inayah], ‘I wouldn't fit in Pakistan…And I wouldn't fit in here. It felt like…I'm halfway between. I'd be too British for Pakistan or too Pakistani for here’ [Mariah], ‘I always felt like I was seen with pity, seen as an outsider’ [Hawa], ‘You just want to fit in with everyone’ [Nadia]. Six participants specifically mentioned how their accent or difference in language contributed to their lack of belonging. Mariah stated, ‘some words I didn't know how to pronounce properly, I had to really work hard to like, get rid of…my accent’, adding that having an accent often highlighted her difference to others.

Additionally, participants acknowledged the lack of Islamic education within the British curriculum, ‘I feel like other ethnicities don't know about the religion, let alone knowing that there's different sects’ [Ameera]. This lack of understanding led to participants feeling a responsibility to educate others, particularly on Shia Islam. Zara explained, ‘I was the one showing people…Muslims, we're not all submissive women. It doesn't mean that if you wear a hijab, you can't do anything’, ‘As an outward Muslim…you feel that weight of responsibility that…I am representing’. Zara further expressed an aspect of ignorance from British people and feeling a duty to correct this. Furthermore, the minority of Shia Muslims created further adversity for Moe; ‘compared to the general population of the university…there's basically one of me, maybe two other Shias…so naturally, I faced a lot of questions’.

#### Conflicting cultural values

This subtheme contributed to participants' sense of not belonging, as aspects of British culture created confusion on their own understanding of their origin culture. ‘I think I was ashamed of my culture, like with food…I didn't really like the school dinners…but I felt embarrassed to bring my own food’ [Mariah]. However, three participants viewed the conflict as a process of growing up. Moe stated, ‘I wouldn't really blame the country or culture…that is just more of a process of growing up’.

Participants mentioned feeling discomfort with aspects of western culture and feeling unaccounted for in England, causing further conflict. Iqra noted the absence of World Hijab Day celebrations at her daughter's school, and Adam expressed the lack of Eastern perspectives in educational settings, ‘I think all of the health professions are taught in a secular way’. Particular discomfort was noted regarding the British drinking culture. Zara shared, ‘Here it's such an alcohol‐y kind of society, it's hard to assimilate or integrate’, and Ali said, ‘if other people are drinking alcohol, I just couldn't relate to them. It was a challenge’. Ahmed also expressed frustration with exclusion due to alcohol; ‘this alcohol at the table restriction I find very…frustrating because simple things like gala dinners…you have to push back’.

The variation in generational statuses between participants was highlighted through conflicting cultural values, as differences were noted. Many second‐generation participants discussed the struggles of helping their parents to understand their decisions after moving to England. Adam explained, ‘I found it really difficult to have to explain to my parents every time I was doing something that we wouldn't have done back home, but it's actually very OK to do here’. Nadia noted, ‘you can still see a bit of a difference, especially between generations…younger generations have definitely taken more British culture and values’. For first‐generation participants, they shared the influence of culture conflict when raising their children. Sarah noted, ‘we've brought them up with a mixture of Islamic and western values…in Somalia it would be different…’. Moreover, Mariah reflected on the isolation through being a first‐generation immigrant, stating, ‘although there was a lot of brown people with similar cultures…they were born here… their British Asian identity was very different to mine as a first‐generation immigrant’.

#### Who am I?

Identity was discussed across the majority of participants. Mariah and Alaina both noted that their British identity was stronger when they were younger, but as they grew older, they started to ‘step back’ [Alaina] and reevaluate who they were. Ahmed further shared his identity challenges; ‘when it comes to identity…there's always a bit of a struggle to know who I actually am…’. He expressed a desire for his children to feel more confident in their identity; ‘with kids I have now growing up…I'm conscious that we need to also ingrain the identity in them as well, so they feel confident to be themselves’. Iqra shared this view when it came to her children; ‘we moved areas and schools so that the kids were not the only Muslim kids…’.

### The emotional toll of acculturation

All participants spoke about the emotional toll of acculturation and its impact on mental health, with isolation being a large contributor. The majority shared feelings of loneliness, sadness and anxiety. Alaina stated, ‘definitely the first couple of years were very difficult for me…the whole thing about not having roots, having to start from scratch…’, and Hawa noted, ‘I was very depressed my first year’. There was a sadness due to migration which was shared, with Sarah expressing that she ‘was missing home…my parents, friends, siblings’. Ali mentioned finding this experience ‘mentally exhausting’, and noted a lack of mental health awareness, sharing, ‘there's still a long way for mental health awareness within the community’. Three participants also shared how bullying impacted their mental health, and others spoke of a lack of acceptance from others around being a Shia Muslim.

Ahmed shared frustration in relation to his appearance, ‘why do I have to have black hair and brown skin rather than being a white person with blonde hair…I thought if I dyed my hair…I'll be more like a white person and receive more…positive attention’. Moreover, six participants felt a sense of guilt due to migration, further impacting their mental health. Ameera explained, ‘I felt so guilty because I knew my parents were doing all of this for me’. Mariah described the pressure she felt to perform, ‘my parents sacrificed so much to be here, I always felt I had to perform‐perform‐perform’. Zara recognised that others would not understand these sacrifices and guilt, further contributing to her isolation.

#### Negotiating authenticity

Participants shared struggles with the emotional toll of living a ‘double life’, understood as code‐switching, for example, between home life and social/school life. Zara recalled feeling overwhelmed by the double life and code‐switching, saying, ‘I wished I was dead before I went to secondary school…it was hard doing the whole double life’. Jawad described the confusion, saying, ‘who do I identify with? In every split there are more and more and more splits. I think with identity it's made a few problems’. Ahmed spoke further, ‘identity is always a tricky one…you're always a different person… at home, your kind of yourself…then at school you're…trying to be British, so you'll speak more with an English accent to…fit in with whoever's around you’. Zain also mentioned the risk of bullying if one did not adjust their language to become more ‘streetwise’ at school.

This constant negotiation created feelings of inauthenticity, stress and isolation. These experiences also extended into therapeutic spaces, where participants expressed hesitancy to discuss the emotional toll of acculturation without the assurance of cultural understanding. More on this can be found in the subtheme below.

#### Seeking culturally safe spaces

This subtheme highlighted the desire for mental health support which is culturally and religiously appropriate. Most participants expressed a preference for therapists who shared their culture or religion. ‘If they're Muslim, it just means they understand the issues more’ [Iqra], ‘it would be better if it is a Muslim…because they can understand the culture’ [Sarah]. Zara shared, ‘it's always nice when you're in that vulnerable position to have someone…if there's something you have in common…you would open up more…feel that you could be honest’.

However, more than half of participants emphasised the importance of confidentiality and anonymity in therapy, explaining that the therapist should not be a member of their own mosque, ‘as part of the community you're cutting out the confidentiality and being anonymous…those are two very important things’ [Jawad].

### Negotiating faith in a secular society

All participants spoke positively of their relationship with religion and the pride it brought to their identity, with Ameera explaining that religion helped her to stay grounded. This pride extended to being a Shia Muslim, ‘for me being Shia is the core foundational part of my identity, there is nothing that supersedes it’ [Moe]. However, sharing this aspect with others, Muslim or non‐Muslim, was difficult for participants. Ahmed noted, ‘I wouldn't volunteer that I'm Shia or I'm different…we just hang around acting like a regular Muslim person’. Hawa also shared this hesitation, ‘I didn't want anybody to see me differently because of being Shia’. Ameera spoke further to this hesitancy, stating, ‘the western world just makes it so hard…I wish I lived in like a Muslim country’.

Participants also spoke about difficulties related to negotiating faith in a predominantly secular society. Adam observed the negative connotations of secularism, explaining, ‘in a secular society, where there's an absence of God…it doesn't mean there's a complete absence of ethics, but it's more influenced by a secular worldview…’. Jawad highlighted the challenges of practicing religion without a top‐down initiative in England, stating, ‘it's a lot easier to just become non‐religious’. He also expressed concerns about religion and culture fading over time, with Nadia adding ‘you have to work that little bit harder to…establish your faith’.

Despite these challenges, participants reflected on the positives of being religious in England. Adam noted that in a less religious society, religion becomes ‘more of a conscious decision and therefore can be a more sincere engagement’. Ali and Hawa also shared that their understanding of religion in England was better than in their origin country, supporting them to become more open‐minded. Many participants explained that being religious in a non‐religious environment deepened their faith. Jawad noted religion shifting from ‘I don't wanna say blind belief because that's important too, but shifting to more logical intellectual thinking, debating, talking’, and this process helped to deepen his faith. Nadia explained, ‘it made me more cognisant of the “why” rather than just doing it’, with Zain agreeing, ‘the challenge has actually made us more attached to the religion’.

### Living with layered discrimination

Participants shared experiences of both covert and overt discrimination in England. Nine participants spoke about covert discrimination, often in the form of ‘odd looks’ [Zain] or ‘multiple stares’ [Ameera]. At times this felt more intense, with Iqra sharing, ‘they literally stare at me from one side of the road all the way one way…then they'd turn their head again and stare’. Several noted experiencing discrimination at work and questioned the possibility of unequal opportunities. Adam shared, ‘consistently there were times where…white colleagues were getting opportunities…there was a repeated pattern of not being favoured’. Four other participants noted similar experiences but worried about how this could be evidenced and whether others would label these incidents as discriminatory, which led to hesitancy in escalating further.

Participants also discussed the challenges of being visibly Muslim, particularly participants who wore the hijab. Hawa shared, ‘I‐I really just thought I was going to get stabbed…I've seen videos of getting your hijab pulled off’. Inayah spoke about the constant stares she received wearing the hijab in England, saying, ‘you never really notice until you go to Muslim country, and it doesn't happen’. Alaina shared wearing hijab when travelling to attend mosque, stating, ‘I'm…very visible. And the visibility will attract negative attention’. Furthermore, Adam and Zain expressed concern for their daughters wearing the hijab.

Participants also recounted overt racism, including being shouted at on the street or having eggs thrown at their houses. Some participants viewed such overt racism as a thing of the past, while others encountered it recently. Despite the seriousness of these experiences, participants recounted them in a relatively casual manner. Inayah described, ‘we did definitely face quite a lot of casual discrimination…people looking at each other…making the odd comment’.

#### Shia‐specific discrimination

The majority of participants described facing discrimination specifically due to being a Shia Muslim. Inayah, Mariah, Moe, Nadia and Zara recounted losing friendships after revealing their Shia identity. Mariah shared, ‘I remember in school somebody was saying something about Shia's being khafir's (non‐believers) …I said I am one…and they cut me off from their friendship group…’. Jawad spoke about being sidelined for being part of a minority within a minority, and Inayah mentioned the fear and trauma she experienced from hiding her Shia identity. Some participants also reported seeing prayer stones, or *turbah's*, which are generally used by Shia Muslims, being moved or discarded in prayer rooms at universities and hospitals. Nadia explained, ‘there would always be issues with turbah's getting thrown in the bin’.

Discrimination from other Muslim sects was highlighted as a significant issue. Participants noted that it was often Muslims from other sects who were discriminatory towards Shia. Jawad felt that this division stemmed from migration, saying, ‘I think there is a division happening after we've moulded into British society’. Inayah also observed that Muslims from other sects were often more discriminatory than non‐Muslims, commenting that ‘white people are not even being as discriminatory as our own people’. However, a few participants expressed feeling fortunate to be in England, as they felt the sectarian divide was even stronger in countries like Saudi Arabia and Pakistan, ‘I've had a lot of trauma…we've actually had a family friend be martyred. He was shot dead just for being Shia…it's been crazy in Pakistan’. [Alaina].

### Holding each other up: Sources of strength

The most profound protective source of strength, shared by all participants, was the importance of having a strong, Shia Muslim community. Ameera highlighted that when choosing her university, the presence of a Shia community nearby was essential; ‘…if I were to move anywhere it would be a place that was centred around religion and had a good community’. Her father also encouraged this. Sarah agreed with this, emphasising that community is vital for her son, ‘I believe 100% community is very important…my son is planning to go to university…all I advise…I want to the community to be very close’. Zain shared that his community played a significant role in his move to England, especially when his father was hospitalised, as they funded his plane ticket, ‘people from the community…helped us find a school…they took us for medicals, for all the relevant interviews, for the vaccinations…’.

For most participants, the strong sense of community provided support and relief during acculturative hardships. Jawad noted, ‘it gives you a place to go to at times of need’. Ali shared a similar sentiment, saying, ‘our community people who used to live nearby…they offered to support us…Alhamdulillah (thank God) …it made a massive difference that somebody was sort of looking after us’.

Another source of strength was the diversity in England, which supported participants in feeling less aware of cultural differences and more accepted. ‘…Coming here, seeing a diverse range of Muslims, it's not just people of my skin colour…it's all‐all kinds of people from all over the place…I've heard the most beautiful stories…understanding that acculturation doesn't always have to negative’ [Alaina]. Iqra expressed that she moved her kids to a school where there was more diversity as her and her husband were finding it challenging to fit in; ‘There was one brown kid, there was one black kid, and all white kids.…my husband said…I don't fit in with these parents…how can I expect my son to fit in?’. A diverse environment led to greater acceptance of cultural differences, as Nadia noted, ‘the fact that my university was so diverse…there were so many other people like me… I think also people were a lot more tolerant…people didn't care about things like hijab’.

Furthermore, Sarah expressed gratitude for the freedom to openly express identity in England, ‘Alhamdulillah (thank God) we are lucky in UK that we can express our identity, we can express our culture, our religion openly’. With this freedom, Moe shared his positive experience, ‘with freedom of expression and thought…I think that has greatly impacted my view on religion in general, because I‐I feel like I am more open to dialogue and discussion with different viewpoints and religions and perspectives’. This sense of openness was seen as a benefit of acculturation.

## DISCUSSION

### Summary of findings

This aim of this research was to explore and gain a deeper understanding of Shia Muslims' acculturative experiences living in England and the impact this has on mental health. To achieve this, 15 participants were interviewed. The primary overarching understanding of participants' experience was ‘navigating through multiple worlds’. This overarching theme emphasised that each participant's journey involved fluid movement across five themes, demonstrating the dynamic nature of their experiences and evidencing Schwartz et al.'s (2010) model of acculturation, reflecting multidimensionality. The five themes understood were: do I belong here, the emotional toll of acculturation, negotiating faith in a secular society, living with layered discrimination and holding each other up: sources of strength.

### Clinical context

The themes identified in this research clearly highlight the challenges faced by Shia Muslims during acculturation, including feelings of exclusion and belonging, experiences of discrimination and struggles with cultural differences. These experiences align with existing literature on the broader Muslim population in England. For instance, a discourse analysis by Anjum et al. (2019) revealed similar findings regarding participants' sense of belonging and identity, noting conflicts between their host culture and culture of origin. British social practices were often seen as directly conflicting with participants' cultural values, thus shaping their acculturative experiences (Anjum et al., 2019). Similar accounts were identified within this research through the subtheme of conflicting cultural values. Furthermore, these experiences align with Schwartz et al.'s (2010) multidimensional model of acculturation, as the conflicting cultural values represent the negotiation of identifications between someone's country of origin and the receiving country.

Additionally, the generational differences observed in this research resonate with Anjum et al.'s (2019) findings. Second‐generation participants were more likely to identify with British culture, while first‐generation participants maintained stronger connections to their origin country. As a result, first‐generation participants often experienced greater acculturative stress. This trend has also been observed in Australian acculturation research, where second‐generation Australian Muslims reported higher levels of assimilation to Australian culture than the first‐generation participants (Abbas et al., 2018). An interesting observation noted in Anjum et al.'s (2019) findings, not noted in this research, is the use of rationalisations participants described when understanding their degree of belonging to a country. Anjum et al. (2019) argue that rationalisations are a way of participants managing the accountability of how and where they belong. This presents a further complex phenomenon when considering identity and belonging through acculturation, differing to these results. This may not have been identified in these findings due to the recruitment being through mosques, providing an anchor in participants' sense of belonging. Therefore, rationalising in the same way as Anjum et al.'s (2019), participants may not have been necessary.

Numerous studies also explore the impact of racism and discrimination within Muslim acculturative experiences (Awad, 2010; Goforth et al., 2014; Kunst et al., 2015; Tineo et al., 2021; Yazdiha, 2018). Furthermore, the Shia‐specific discrimination highlighted by participants is a phenomenon documented in the literature, though not within UK‐based research. Rather, as noted in the introduction, it has been observed in countries such as Pakistan (Syed & Ali, 2020), Saudia Arabia (Watch & Wilcke, 2009), Malaysia (Musa & Tan, 2017) and the broader Middle East (Salnikov, 2019). Despite these findings, limited literature remains exploring the impact of this specific form of discrimination on Shia Muslims' acculturative stress, highlighting an important area for future research.

A noteworthy observation when reviewing the existing literature on Muslim acculturation is the minimal positive accounts of this process. This research revealed several sources of strength through the diversity and sense of community within England. Often, participants expressed gratitude for the available resources, contributing to freedom in identity and expression. Much of the literature speaks of the challenges faced during acculturation, for example, acculturative stress (Berry, 2007), isolation (Bagby, 2010; Toki et al., 2023) and discrimination (Nakash et al., 2012). Schwartz et al.'s (2010) model states that acculturation is influenced by sociocultural environments, providing room for protective factors of the acculturative process.

This study highlights the importance of recognising the sources of strength that can support individuals to navigate and thrive through acculturation, such as community support. This has further been identified in the acculturative literature. Arnold and Schneider's (2007) research looked at the role of how ethnic online communities create cultural identity. In their sample of Turkish participants living in Germany, it was important for individuals to connect with those who had similar living conditions and were of the same ethnic and cultural roots. These platforms were seen as channels for participants to strengthen their Turkish cultural identity. This research demonstrates how community support can reduce isolation for those who are undergoing acculturation.

Existing literature has also reported on the importance of faith through acculturative experiences, as participants have echoed the strength and support Islam has provided for them (Akram, 2012; Hussain, 2024; Khuwaja et al., 2013; Tineo et al., 2021). When looking at this through Schwartz et al.'s (2010) model, we can see the importance of faith‐based values and the impact of these belief systems remaining strong through acculturation.

It has been helpful to keep in mind Schwartz et al.'s (2010) multidimensionality of acculturation model in this research. It has allowed for the appreciation of the complex processes of participants acquiring culture through acculturation, but also juggling the retainment of their own heritage culture. Schwartz et al. (2010) argue that acculturation is not a singular process that takes place at a singular pace. This is reflected through the thematic map (Figure 2), which represents acculturation as a journey that participants navigate through various themes. Acculturation is multidimensional, that needs to account for someone's identity, their values and practices (Schwartz et al., 2010).

### Clinical implications

The findings from this research suggest that the path to acculturative adjustment for British Shia Muslims is complex, fluid and can be emotionally draining. Their journey often involves feelings of isolation, cultural confusion and discrimination, all of which have a direct impact on mental health and wellbeing.

Based on the findings, the first recommendation is ensuring therapeutic workforces within the NHS reflect the diversity of the communities they serve, as participants expressed a preference for practitioners who shared their religion or culture. This is supported by Field and Caetano (2009), who argue that ethnic harmony and a shared cultural understanding between patients and practitioners lead to more effective interventions, due to better recognition of cultural factors. Moreover, a multicultural workforce has shown to yield better outcomes, including higher psychological engagement, reduced perceived bias and reduced job turnover within racial‐ethnically minoritised groups (Gündemir et al., 2019). Hussain et al. (2020) further emphasise that achieving an ethnically diverse workforce requires intentional effort, including ethnically centred recruitment strategies, dedicated resources and thoughtful planning to ensure meaningful representation.

However, challenges could be associated with the recommendation of diversifying the therapeutic workforce. Therefore, further implications need to be considered such as the education and development of allied health professionals during their training. Participants noted that the British population generally did not understand Islam, which exacerbated their feeling of not belonging. Furthermore, Islam played a fundamental role in participant identities. Dein et al. (2010) noted that religious considerations are often overlooked in mental health assessments, though including them can lead to better outcomes. Therefore, practitioners could consider how they inquire about religion or spirituality in assessments. Models such as Garraway's (2016) CBT‐based approach which involves the spirit, or Borneman et al.'s (2010) FICA model, which encompasses Faith, Importance, Community and Addresses how the individual would like this to be involved in their care, can be effective. These approaches may help practitioners to feel comfortable asking about the role of religion and/or spirituality in someone's life. Additionally, the complexity of being a Shia Muslim due to existing as a ‘minority within a minority’ (Scharbrodt, 2019) was noted in these results. This highlights the need for open models that allow individuals to choose the level of detail they share about their religious experiences, which can be done through Garraway (2016) and Borneman et al.'s (2010) models.

The conflicting cultural values that participants shared through acculturation can inform a further clinical implication. Cultural competence and cultural humility are essential when working with minoritised communities. Gulati and Weir (2022) emphasise the importance of fostering cultural reflection and encouraging discussions in cultural humility throughout training. They argue that cultural competence is not only morally imperative but also enhances quality of care. Rice and Harris (2020) further highlight that cultural competence improves comfortability within health care settings, and with practitioners having a better understanding of the cultural difficulties minoritised participants experience, this may support them to feel better understood.

Despite the similarities noted through religion, culture and discrimination for participants, as highlighted in the introduction, it is essential to remain attentive to the intersectionality of all individuals. This is a fundamental aspect of working psychologically (Tang et al., 2020) as individuals are multidimensional. This can be seen through the generational differences that were shared by participants when it came to acculturative experiences. McCormick‐Huhn et al. (2019) highlight the importance of adopting an intersectional lens, noting that it provides a deeper understanding of how power interacts with an individual's experience. These results have demonstrated specifically how appreciating an individual's religion and culture can be therapeutically helpful. Being aware of additional identities that someone holds can enable a more effective, efficient approach (Butler, 2017), and this can be done with the aid of the other Social GRACES (Burnham, 2018).

Finally, the layered discriminatory experiences noted in the results are important to reflect upon when supporting this population. This is particularly important in light of the current political climate, including the recent Islamophobic riots in England. Islamophobia and racism directly impact Muslim mental health by fostering a sense of stigma, leading to internalised feelings of low self‐esteem, hopelessness and discrimination (Bhui et al., 2024). The final recommendation to be considered is developing the communication between services and communities, so that minoritised groups are aware of the steps being taken by services to ensure inclusivity and the implementation of intersectionality. This approach would also help in developing religious literacy for mental health practitioners (Abrar & Hargreaves, 2023). A dearth of literature was identified on assessing the communication between minoritised communities and service providers. This may be because it is not a heavily researched area, or that methods of communication have not yet been developed. However, future research is vital to support services in building these lines of communication. Strengthening communication between services and communities could promote a greater sense of safety for ethnically minoritised communities, especially those discriminated against.

### Study limitations and further research

Many limitations of this project stem from the participant pool and recruitment process. The results highlighted that the mosque and community were central to participants' lives, including moving to areas with a known Shia community presence. This commonality among participants may have related to the recruitment from mosques. Additionally, most participants had a baseline level of religious belief which could have also been related to mosque recruitment, further narrowing the scope of experiences. The existing research on the relationship between religion and acculturation (Awad, 2010; Güngör et al., 2013; Niens et al., 2012) highlights the protective and supportive role that religiosity can provide during times of acculturative hardship (Güngör et al., 2013). Consequently, this recruitment procedure led to a more constrained participant pool, with greater emphasis on religiously observant individuals. Those who are not as closely affiliated with mosques, and perhaps religion, may have other factors that become significant through acculturation, which were not captured. It is important to note that the goal of this research was not to generalise the findings as the interpretivist approach emphasises the uniqueness of each individual's experience. Nonetheless, further research exploring acculturative experiences in Shia Muslims not closely associated with a mosque could further develop understandings around the topic.

Another limitation of the participant pool was the lack of variation in generational status. Despite the recruitment poster specifying a desire to include first‐, second‐ and third‐generation participants, all either fell into first or second‐generation categories. Furthermore, all participants were English speaking, with 93% identifying English as their first language. As a result, the findings are limited to these specific contexts. The fact that participants were English speaking indicates that a certain level of acculturation has occurred, as Brown (1994) notes that language and culture are intrinsically linked. The relationship between language and culture is complex, and separating the two can diminish the significance of either one (Brown, 1994). Therefore, it would be interesting to explore whether the results and themes would differ with non‐English‐speaking participants, who may face different acculturative challenges. For example, previous research has found that Chinese immigrant students who experience discrimination due to language barriers are more likely to prioritise maintaining their own cultural heritage over assimilating to the host culture (Gu et al., 2021). This presents an area for future research, where the experiences of non‐English‐speaking participants could offer further valuable insights into the additional dimensions of acculturation.

## CONCLUSION

The findings of this research highlight the complex and challenging experiences that British Shia Muslims face during acculturation. It is hoped that these results will contribute to the development of intersectional thinking within mental health services, emphasising the need for a deeper understanding of the Shia Muslim community. The clinical implications to support this are diversifying the workforce, enhancing training to allied health professionals, fostering cultural competence and humility, adopting an intersectional approach and being aware of and addressing discrimination. Finally, future research should attempt to recruit without mosque affiliations and consider engaging non‐English‐speaking participants to gain a broader understanding of the challenges faced by these communities.

## AUTHOR CONTRIBUTIONS

**Mahdiyah Datoo:** Conceptualization; methodology; writing – original draft; writing – review and editing; data curation; investigation; visualization; formal analysis. **Sanaa Kadir:** Writing – review and editing; supervision; conceptualization; methodology.

## FUNDING INFORMATION

Funding was received as part of my DClinPsy by the University of Leicester. No other external sponsorship or funding was involved.

## CONFLICT OF INTEREST STATEMENT

The authors declare no conflicts of interest.

## Data Availability

Research data are not shared.
